# Alternate quantification approaches for cold-induced vasodilation in human glabrous skin

**DOI:** 10.3389/fphys.2025.1575764

**Published:** 2025-06-10

**Authors:** J. A. Stout, D. E. Gerow, P. C. Clegg, K. Metzler-Wilson, T. E. Wilson

**Affiliations:** ^1^ Indiana University School of Medicine, Indianapolis, IN, United States; ^2^ Marian University College of Osteopathic Medicine, Indianapolis, IN, United States; ^3^ New England Baptist Hospital, Boston, MA, United States; ^4^ Novant Health Rehabilitation Hospital, Winston-Salem, NC, United States; ^5^ Department of Physical Therapy, Indiana University - Indianapolis School of Health & Human Sciences, Indianapolis, IN, United States; ^6^ Department of Anatomy, Cell Biology, & Physiology, Indiana University School of Medicine, Indianapolis, IN, United States; ^7^ Department of Dermatology, Indiana University School of Medicine, Indianapolis, IN, United States; ^8^ Department of Physical Therapy, University of Kentucky College of Health Sciences, Lexington, KY, United States; ^9^ Department of Physiology, University of Kentucky College of Medicine, Lexington, KY, United States; ^10^ Saha Cardiovascular Research Center, University of Kentucky College of Medicine, Lexington, KY, United States

**Keywords:** fast-Fourier transform, transfer function analysis, laser Doppler flowmetry, sympathoexcitation, functional sympatholysis

## Abstract

**Introduction:**

Cold-induced vasodilation (CIVD) is a counterintuitive focal increase in glabrous skin blood flow during cold exposure with unclear local and neural mechanisms.

**Methods:**

We tested 12 (8 men, 4 women) healthy subjects’ laser-Doppler flux (LDF; just proximal to the nailbed) and arterial blood pressure (ABP) on a beat-by-beat basis. The experimental hand was exposed to warm (10 min 35°C) and then cold (30 min 8°C) water immersion and the contralateral control hand experienced 22°C–23°C air throughout. We analyzed beat-by-beat oscillations in LDF and ABP via a fast-Fourier transform (FFT) and transfer function analysis (TFA) of LDF to ABP.

**Results:**

LDF spectral power was greater in the control finger than immersed fingers in the normalized very low frequency (nVLF) range. There was an interaction in the normalized low frequency (nLF) range where cooling decreased power in immersion sites but increased power in the control site. VLF and LF TFA gains were lower during cooling for immersion but not control sites. Data confirm a significant effect of local vasoconstriction within sympathetic vasoconstriction as identified by changes in VLF and LF, respectively. Comparing CIVD bins (LDF criteria, n = 6) to general cutaneous vasoconstriction bins with no CIVD (n = 6) yielded increased nVLF (P = 0.05) and decreased nLF (P = 0.09) power with CIVD.

**Discussion:**

Thus, the unique analysis of LDF and ABP using the FFT-TFA approach appears to be beneficial in providing insights into CIVD events with a periodic local release of vasoconstriction under varying sympathetic tone.

## Highlights

• Oscillations in arterial blood pressure and skin blood flux were analyzed during finger cooling.
• Cooling increased arterial blood pressure and decreased finger cutaneous vascular conductance.
• Cold-induced vasodilation increased very low and decreased low frequency spectral power.
• Release of vasoconstriction occurs under sympathetic tone during cold-induced vasodilation.

## Introduction

Decreasing heat loss via cutaneous vasoconstriction is a fundamental response to cold stress. In humans, this is mediated by sympathetic noradrenergic vasoconstriction coupled with local temperature factors and paracrine signaling that reduce blood flow to skin areas that interface with the environment (Alba et al., 2019; Johnson and Kellogg, 2018). In the skin of the hands, feet, and face, cold-induced vasoconstriction is coupled with periodic increases in skin blood flow and temperature (cutaneous cold-induced vasodilation; CIVD) during prolonged exposure to low temperatures (Cheung, 2015; Daanen, 2003). Despite observations dating back nearly 100 years (Lewis, 1930) and similar observations in certain skin of several species (Folkow et al., 1963; Gardner and Webb, 1986; Mathew et al., 1978; Meyer and Webster, 1971), the precise mechanisms of CIVD are still unclear.

Several factors contribute to this lack of mechanistic clarity; these include the quantification issues associated with CIVD (i.e., direct vs. indirect measures of skin blood flow), its uneven tissue distribution and heterogeneity, and the lack of occurrence predictability of the response. One anatomical feature that appears to play at least a partial role in CIVD is arterio-venous anastomoses (AVAs) (Bergersen et al., 1999); however, the precise control and regulation of these vessels have yet to be fully identified. One factor that is well correlated to the opening and closing probability of AVAs is arterial blood pressure (ABP) (Walloe, 2016). The cold and cold pain sensations that are often concurrently experienced in cold water immersion increase sympathetic activation and ABP (Cui et al., 2002; Metzler-Wilson et al., 2020), likely via a transient receptor potential ankyrin type 1 (TRPA1) mechanism. TRPA1 has also been implicated in cold-induced responses: a) local vasoconstriction via TRPA1 on vascular smooth muscle and b) local vasodilation via TRPA1 on sensory afferents possibly releasing a substance to relax vascular smooth muscle (Aubdool et al., 2014). Thus, investigations of CIVD may benefit from assessment using indices of sympathoexcitation and ABP as part of measured parameters.

Analytical techniques may provide insights into the mechanisms of oscillatory CIVD responses. These techniques most commonly include: a) wavelet analysis and b) fast-Fourier transform (FFT) coupled with transfer function analysis (TFA). Wavelet analysis is an easy-to-use and efficient signal processing approach which allows for use of variable time bins but which only utilizes a single parameter. Proponents have asserted some elegant potential mechanisms relating to various frequency bins (Stefanovska et al., 1999); however, many of these bins have neither been verified for independence (i.e., a perturbation that affects one frequency bin also affects other bins) nor extensively interrogated for mechanism. Several investigators have used this approach to identify further decreases in the 0.02–0.05 Hz frequency band during CIVD than the decrease already observed in cold-induced vasoconstriction (Hodges et al., 2019; Hodges et al., 2018; Sera et al., 2020). Using the FFT-TFA approach, via human ganglionic blockade and reintroduction of vascular pressure waves, Wilson et al. (Wilson et al., 2005) identified that both low frequency (LF; 0.07–0.2 Hz) and very low frequency (VLF; 0.02–0.07 Hz) oscillations in skin blood flow are mediated and affected by the sympathetic nervous system. This indicates that frequency bins may be less mechanistically independent than originally proposed. The FFT-TFA approach is used in cardiovascular research for heart rate, ABP, and vascular autoregulation (Panerai et al., 2023; Zhang et al., 2002a), due to its greater quantification of sympathetic effects (as it has been used with agonists, antagonists, and in controlled comparative models) and the ability to relate ABP to blood flow. While FFT-TFA allows for better resolution at very low frequency ranges and has the advantage of allowing for incorporation of ABP, which drives skin blood flow, this approach has not yet been employed in CIVD research.

This study aims to more precisely identify the sympathetic and ABP components of the CIVD response. We employed a) beat-by-beat measures of pressure and flow to improve CIVD quantification via time series data and spectral power by frequency data using FFT-TFA, b) skin erythrocyte concentration (to gain insight into vascular volume), and c) classical skin temperature parameters in multiple fingers. We hypothesized that 1) FFT and TFA will provide a CIVD signature compared to cold segments without a CIVD. 2) CIVD will have a significant effect on VLF and LF components of FFT. 3) CIVD will have increased transfer function gain compared to vasoconstriction without dilation.

## Methods

### Subjects

Twelve healthy subjects (8 men and 4 women; self-identified) participated in the study. We used both health history questionnaire and normal vital signs (oral temperature 36.5°C–37.5°C; systolic <130 mmHg, diastolic <90 mmHg, and mean ABP; heart rate 60–100 bpm; and pulse oximetry >95% [Dinamap V100, GE Healthcare, Chicago, IL, United States]) to determine the health status of the participants before their inclusion. Participants were not on any medications, nor were any participants smokers or smokeless tobacco users. We asked participants to refrain from caffeine, and vigorous exercise was prohibited for 12 h prior to testing. The participants’ average (SD) age, weight, height, body mass index (BMI), and body surface area were as follows: 25 (3.5) years, 76.9 (15.0) kg, 176.0 (8.6) cm, 24.6 (3.2) kg/m^2^, and 1.9 (0.22) m^2^. The Marian University Institutional Review Board approved both the experimental protocol and the informed consent process. We obtained verbal and written consent from each of the participants before the commencement of the experiment, and the study complied with the most recent version of the Declaration of Helsinki.

### Measurements

We measured blood pressure non-invasively on a beat-by-beat basis via finger photoplethysmography (Finometer Pro, FMS, Amsterdam, NLD). We took this measurement by placing the blood pressure cuff on the right 4th finger. We placed laser-Doppler flowmetry integrated probes (LabFlow, Moor Instruments, Devon, GBR) proximal to the nailbeds of the left 2nd and 3rd fingers and right 2nd finger to measure skin blood flux and erythrocyte concentration. We placed thermocouples (T-type, Omega Engineering, Norwalk, CT, United States) on the finger pads of the corresponding fingers and connected them to a meter (TC-2000 Sable Inst., Las Vegas, NV, United States) to monitor skin temperatures. An electrocardiogram (BMA-400 Bioamplifier, CWE Inst., Ardmore, PA, United States) and ModelFlow analysis of the arterial waveform (BeatScope, FMS) enabled cardiac output and stroke volume calculations.

### Experimental protocol

**FIGURE 1:**
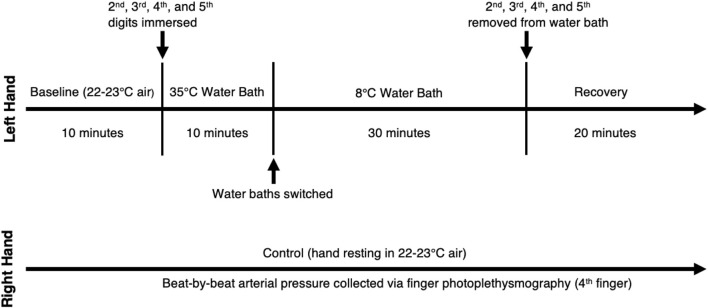
Protocol Schematic. Left hand is the experimental hand and right hand is the control hand.

After instrumentation, we instructed the participants to rest for 10 min to establish a baseline (22°C–23°C). Participants then submerged the digits of their left hand (excluding the thumb) to the proximal interphalangeal (PIP) joint in a warm-water bath (35°C) for 10 min to reduce vasoconstriction and equalize finger temperatures between subjects. They then submerged the same digits to the PIP joint in a cold-water bath (8°C) for 30 min. Once the cooling period concluded, we dried the hands of the participants and initiated a 20-min recovery period consisting of spontaneous rewarming without movement. The right hand served as a control and rested outside of the water for the duration of the experiment. A schematic of the protocol can be seen in Figure 1.

### Data analysis

We collected all measurements at 100 Hz via a data acquisition system and software (MP 150 & Acknowledge 4.4 BioPac Sys., Goleta, CA, United States). We excluded one of the original 13 participants from analysis due to aberrant skin blood flow readings. We calculated cutaneous vascular conductance (CVC) by dividing laser-Doppler flux (LDF) by mean arterial pressure, using 5-min time bins. We used the last 5 min of the baseline and warming portions of the protocol in calculating baseline values and warming values, respectively. For the cooling portion of the protocol, we used the first 5 min in determining the initial cooling values and the last 5 min of the protocol in finding the final cooling values.

We performed frequency analyses of beat-by-beat ABP and the LDF of two test fingers and the control finger during baseline, warming, initial cooling, and final cooling periods using a fast-Fourier transform (FFT) to identify power spectral density (Acknowledge 4.4, Biopac & DADiSP 6.0, DSP Development Corporation, Newton, MA, United States). First, we detrended the time series of mean skin blood flow and mean arterial pressure using third-order polynomial fitting. We then divided into 256 point segments with 50% overlap, yielding 602 segments over 5-min bins. We averaged the Hanning-windowed data segments to obtain a spectrum. We binned the power spectral density according to physiological frequencies that have been previously described in the systemic cardiovascular system and in specific vascular beds (Cooke et al., 2002; Zhang et al., 2002b) and normalized to total power. These bins were very low frequencies (VLF; 0.02–0.07 Hz), low frequencies (LF; 0.07–0.20 Hz), and high frequencies (HF; 0.20–0.35 Hz). We also performed transfer function analysis (TFA) of LDF of each finger to ABP to obtain gain, phase, and coherence between these biological signals. We performed the above analyses for 9 participants, as we were unable to analyze beat by beat parameters in the other participants due to ECG artifact. Finally, as a subset analysis, we compared timepoints with and without LDF CIVD in 6 participants who had distinct CIVD vs. non-CIVD bins during cold water immersion.

To assess ABP, R-to-R interval, stroke volume, and cardiac output, we used one-way repeated measures ANOVA to determine whether differences existed across timepoints (baseline, warming, initial cooling, and final cooling). To assess skin temperature, LDF, CVC, erythrocyte concentration, and the absolute and relative mean spectral powers of LDF signals, we used two-way repeated measures ANOVA to determine whether differences existed between conditions (control and test fingers) and across fixed timepoints (air baseline, warm water immersion, and initial and final cold water immersion). For the analysis of CIVD vs. non-CIVD power spectral density, we used one-way repeated measures ANOVA to determine whether differences existed between incidences (CIVD and no CIVD). We conducted a post-hoc Student-Newman-Keuls analysis when significant main effects were found. For the baseline vs. warming comparisons of power spectral densities, we used 1-tailed t tests for each finger. We set statistical differences at α ≤ 0.05 and statistical trends were set at α ≤ 0.10 but >0.05. Effect sizes were calculated and presented with statistical probabilities. Partial eta squared (*η* ^2^) was used for interactive and main effects and Cohen’s d (*d*) for pairwise comparisons.

We identified CIVD, as defined by characteristic temperature fluctuations, via visual inspection conducted by two blinded reviewers. We defined this response as an increase of greater than 1°C from the minimum temperature following submersion in the cold water (Hodges et al., 2018). We also determined CIVD by inspecting the LDF associated with the 2nd and 3rd digits of the test hand. This assessment was based on qualitative measures; the reviewers deemed a participant to demonstrate a response if there was a clear, sustained upward slope in the LDF tracing following its minimal value during initial vasoconstriction (O'Brien, 2005). In cases of initial disagreement, the reviewers performed additional inspection and discussion until they reached consensus.

## Results

### Systemic cardiovascular responses

Partial hand cold water immersion increased both mean arterial pressure and R-to-R interval durations. In contrast, both stroke volume and cardiac output were unaffected by partial hand cold water immersion (Table 1).

**TABLE 1:** Effects of the experimental procedure on systemic responses.

Systemic response	Air baseline	Partial-hand water immersion	
	22–23°C	35°C	8°C
Arterial Blood Pressure (mmHg)	85 ± 4	84 ± 4	95 ± 4*
R-to-R Interval (ms)	0.82 ± 0.07	0.87 ± 0.07*	0.89 ± 0.07*
Stroke volume (mL)	97 ± 6	97 ± 6	99 ±5
Cardiac Output (L/min)	7.1 ± 0.6	6.6 ± 0.6	6.5 ± 0.5

The values indicate the means ± SE. * indicates significant difference from baseline.

### Erythrocyte concentration and skin temperature

**FIGURE 2:**
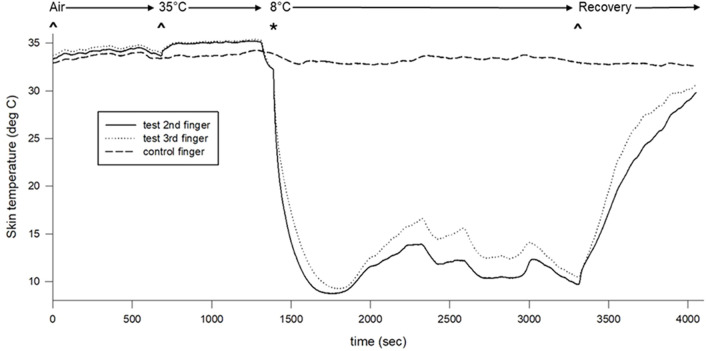
Skin surface temperature changes across the protocol. Air indicates air baseline, 35°C indicates warm water immersion of test fingers, 8°C indicates cold water immersion of test fingers, and recovery indicates the recovery period.

**FIGURE 3:**
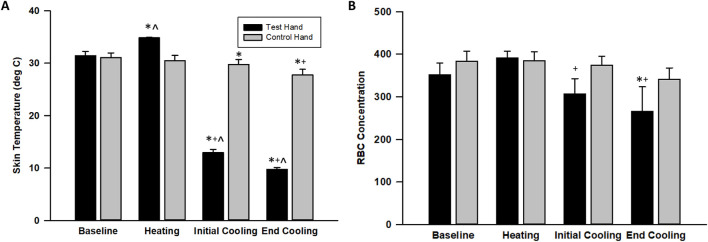
Skin surface temperature **(A)** and erythrocyte (RBC) concentration **(B)** changes across the protocol. The values indicate the means ± SE (n = 9 participants). Heating indicates warm water immersion timepoint and cooling indicates final cold water immersion timepoint. * indicates significant difference from baseline. ^ indicates significant difference from control. ^+^ indicates significant difference from warm water immersion timepoint.

We did not identify differences between test and control hand erythrocyte concentration or skin temperature during baseline. The warm water immersion increased skin temperature in the test hand only, but this increase was not associated with alteration of erythrocyte concentration. The erythrocyte concentration (test hand) and skin temperature (both hands) decreased and remained significantly lower throughout the cooling portion of the protocol. Finally, significant differences occurred between test and control hand skin temperatures throughout the cold water immersion perturbation (Figures 2, 3).

### Cutaneous vascular conductance (CVC)

**FIGURE 4:**
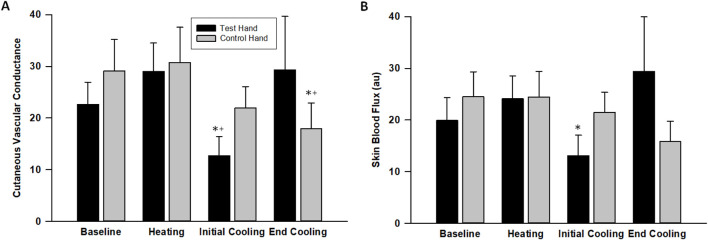
Cutaneous vascular conductance **(A)** and laser-Doppler flux **(B)** changes across the protocol. The values indicate the means ± SE (n = 9 participants). Heating indicates warm water immersion timepoint and cooling indicates final cold water immersion timepoint. * indicates significant difference from baseline. ^+^ indicates significant difference from warm water immersion timepoint.

The CVC in the test hand increased during warm-water immersion by 38% ± 12% (p = 0.007, *d* = 1.32) before decreasing during initial cooling by 48% ± 9%, reaching a value that was lower than its baseline (p = 0.003, *d* = 1.64 Figure 4). By final cooling, however, the CVC in the test hand increased such that it was no longer significantly different from baseline (p = 0.841, *d* = 0.10). The control hand, though, maintained a similar CVC (p > 0.112) until final cooling, during which its CVC was significantly lower than its baseline value (p = 0.006, *d* = 1.51, Figure 4). Skin blood flux showed similar patterns as CVC (Figure 4).

### CIVD response rate

CIVD occurred in 10 of the 12 participants as defined by finger pad skin temperature, while 8 participants exhibited CIVD when defined by nailbed LDF. CIVD descriptive data include: minimum temperature = 8.8°C ± 0.2°C, maximum temperature = 11.5°C ± 0.6°C, amplitude = 2.7°C ± 0.5°C, onset time = 363 ± 42 s, peak time = 434 ± 97 s, frequency = 1.7 ± 0.2 of individuals with at least one CIVD identified.

### Absolute LDF spectral power

In our fixed timepoint (air baseline, warm water immersion, initial and then final cold water immersion) analysis, we observed main effects of timepoint on LF spectral power (p = 0.036, *η* ^2^ = 0.088) and trend towards main effect on HF spectral power (p = 0.067, *η* ^2^ = 0.033). There was a trend in the interaction between time period and condition for LF (p = 0.068, *η* ^2^ = 0.045). The timepoint differences were specifically a decrease in LF spectral power between the warm water immersion and initial cold water immersion (p = 0.033, *d* = 1.01) and between warm water immersion and final cold water immersion (p = 0.048, *d* = 1.04), and a trend towards an increase from air baseline to warm water immersion (p = 0.091, *d* = 0.66) (Table 2, Panel A).

**TABLE 2:** Mean spectral powers of LDF signals for each probe (Panels A and B) and arterial blood pressure (Panels C and D). Panels A and C depict values in very low (VLF), low (LF), and high (HF) frequency ranges and Panels B and D depict normalized values in the same ranges (nVLF, nLF, and nHF) (n = 10). 35°C indicates warm water immersion timepoint and 8°C indicates final cold water immersion timepoint.

Panel A. Skin ‐ absolute values				
Probe	Partial-hand water immersion	VLF (flux units^2^)	LF (flux units^2^)	HF (flux units^2^)
Test hand (2nd digit)	35°C	1,462.69 ± 992.71	921.46 ± 454.87	62.15 ± 30.69
	8°C	38.57 ± 19.72	148.72 ± 122.00*	35.77 ± 23.05
Test hand (3rd digit)	35°C	1,477.99 ± 539.15	1,378.22 ± 486.14	137.15 ± 64.45
	8°C	1,396.23 ± 1,047.86	307.17 ± 171.84*	76.70 ± 43.92
Control hand	35°C timepoint	1,450.06 ± 487.72	866.04 ± 338.41	58.08 ± 17.18
	8°C timepoint	786.17 ± 282.93	582.36 ± 220.69	32.66 ± 11.11

Panel B. Skin ‐ normalized values				
Probe	Partial-hand water immersion	nVLF (flux units^2^)	nLF (flux units^2^)	nHF (flux units^2^)
Test hand (2nd digit)	35°C	52.28 ± 4.69^^^	43.49 ± 4.76^^^	4.23 ± 0.74
	8°C	42.18 ± 5.84^^^*	40.99 ± 5.14	16.84 ± 2.51^^^
Test hand (3rd digit)	35°C	49.07 ± 6.64^^^	46.77 ± 6.35^^^	4.15 ± 0.55
	8°C	47.15 ± 7.65^^^*	38.41 ± 5.70	14.44 ± 2.56^^^
Control hand	35°C timepoint	64.23 ± 7.18	32.76 ± 6.67	3.01 ± 0.64
	8°C timepoint	64.63 ± 5.65	32.34 ± 5.55	3.04 ± 0.76

Panel C. Blood pressure ‐ absolute values				
	Partial-hand water immersion	VLF (mmHg^2^)	LF (mmHg^2^)	HF (mmHg^2^)
Arterial blood pressure (mmHg^2^)	35°C	986.81 ± 125.72	751.90 ± 123.99	176.77 ± 50.73
	8°C	1,041.93 ± 263.39	1,143.27 ± 269.56	262.45 ± 71.97

Panel D. Blood pressure ‐ normalized values				
	Partial-hand water immersion	nVLF (mmHg^2^)	nLF (mmHg^2^)	nHF (mmHg^2^)
Arterial blood pressure (mmHg^2^)	35°C	52.37 ± 3.77	38.76 ± 3.48	8.87 ± 1.86
	8°C	45.96 ± 6.00	44.29 ± 5.09	9.75 ± 1.30

The values indicate the means ± SE. ^ indicates significant difference from control. * indicates significant difference from warm water immersion.

### Normalized LDF spectral power

In our fixed timepoint (air baseline, warm water immersion, initial and then final cold water immersion) analysis, we observed interactions between fingers and timepoint for nVLF (p = 0.018, *η* ^2^ = 0.057), nLF (p = 0.013, *η* ^2^ = 0.066), and nHF (p < 0.001, *η* ^2^ = 0.113). We noted specific decreases in nVLF spectral power in both warm water immersion fingers compared to the contralateral control finger in room air (p = 0.004, *d* = 1.36 and p = 0.001, *d* = 1.64 for digit 2 and 3, respectively) and final cold water immersion (p < 0.001, *d* = 1.78 and 1.74 for both digit 2 and 3). Specific increases were noted in nLF spectral power in warm water immersion compared to the contralateral control finger in room air (p = 0.006, *d* = 1.31 and p = 0.002, *d* = 1.62 for digit 2 and 3, respectively). Significant increases were noted in nHF in both cold water immersion timepoints compared the contralateral control finger in room air (All comparisons p < 0.001, *d* > 1.77, except between cold immersion digits in both timepoints. Comparisons between cold immersion digits were p = 0.366, *d* = 0.41 and p = 0.866, *d* = 0.08 for initial and final cooling, respectively.) (Table 2, Panel B).

### ABP spectral power

ABP spectral power did not change across any timepoint or any frequency, whether expressed in absolute values or normalized to total spectral power (Table 2, Panel C). When normalized, trends emerged for nVLF (p = 0.066, *η* ^2^ = 0.160) and nHF (p = 0.061, *η* ^2^ = 0.159) due to some lower and higher values in initial cooling than the other timepoints, but the variability was too great to achieve significance (Table 2, Panel D).

### TFA of LDF to ABP

**FIGURE 5:**
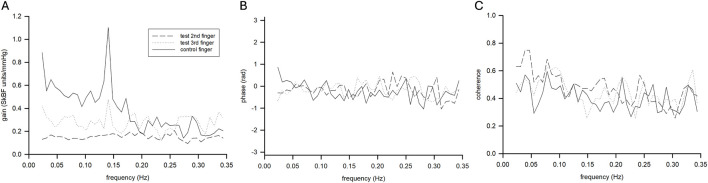
Mean transfer function gain **(A)**, phase **(B)**, and coherence function **(C)** for control (solid line) and test (dashed lines) fingers during the final cooling timepoint (n = 9 participants).

Average pooled TFA coherence between LDF and ABP was 0.5 ± 0.2, 0.5 ± 0.1, and 0.4 ± 0.1 for warm and 0.5 ± 0.2, 0.5 ± 0.2, and 0.4 ± 0.1 for cold for VLF, LF, and HF, respectively. These results satisfied coherence guidelines for use of TFA in the VLF and VF, but not the HF (Panerai et al., 2023). We observed timepoint differences in VLF (p = 0.015, *η* ^2^ = 0.136), LF (p = 0.007, *η* ^2^ = 0.182 and HF (p = 0.018, *η* ^2^ = 0.131), where TFA gain was lower during cooling compared to air baseline and warm water immersion. We observed interactions between fingers and timepoint for LF (p = 0.009, *η* ^2^ = 0.036) in TFA gain. Specific decreases were noted in LF TFA gain in both fingers during final cold water immersion compared to the warm water immersion (p = 0.003, *d* = 3.15 and p < 0.001, *d* = 3.82 for digit 2 and 3, respectively). We observed interactions between fingers and timepoint for VLF (p = 0.022, *η* ^2^ = 0.047) for TFA phase. Specifically, cooling decreased phase (time-delay) of the LDF to ABP signal in the VLF during cooling immersion sites, but the extent was somewhat finger dependent (Figure 5; Table 3). There was a trend for group differences in LF TFA phase (p = 0.054, *η* ^2^ = 0.050), where immersion fingers were less negative than the control air finger (Figure 5; Table 3).

**TABLE 3:** Transfer function analysis of arterial pressure to skin blood flux variability during partial-hand water immersion (35°C and 8°C) for each probe. 35°C indicates warm water immersion timepoint and 8°C indicates final cold water immersion timepoint.

Panel A. Test hand (2nd digit)				
Transfer function analysis	Partial-hand water immersion	VLF	LF	HF
Transfer function gain (flux units/mmHg)	35°C	0.58 ± 0.11	0.74 ± 0.15	0.34 ± 0.04
	8°C	0.14 ± 0.05	0.14 ± 0.07*	0.13 ± 0.05
Phase (radians)	35°C	0.13 ± 0.24	−0.07 ± 0.12	−0.42 ± 0.10
	8°C	−0.13 ± 0.15	−0.23 ± 0.12	−0.11 ± 0.19
Coherence (U)	35°C	0.48 ± 0.07	0.54 ± 0.05	0.39 ± 0.03
	8°C	0.63 ± 0.04	0.48 ± 0.06	0.39 ± 0.05

Panel B. Test hand (3rd digit)				
Transfer function analysis	Partial-hand water immersion	VLF	LF	HF
Transfer function gain (flux units/mmHg)	35°C	0.87 ± 0.17	0.99 ± 0.16	0.50 ± 0.07
	8°C	0.38 ± 0.17	0.29 ± 0.10*	0.28 ± 0.09
Phase (radians)	35°C	0.82 ± 0.27	−0.06 ± 0.13	−0.41 ± 0.10
	8°C	−0.21 ± 0.21*	−0.09 ± 0.13	−0.20 ± 0.13
Coherence (U)	35°C	0.59 ± 0.05	0.55 ± 0.03	0.39 ± 0.04
	8°C	0.48 ± 0.06	0.46 ± 0.06	0.42 ± 0.05

Panel C. Control hand				
Transfer function analysis	Partial-hand water immersion	VLF	LF	HF
Transfer function gain (flux units/mmHg)	35°C timepoint	0.76 ± 0.19	0.69 ± 0.21	0.39 ± 0.10
	8°C timepoint	0.61 ± 0.12	0.46 ± 0.17	0.22 ± 0.05
Phase (radians)	35°C timepoint	0.64 ± 0.24	−0.23±-0.15	−0.16 ± 0.14
	8°C timepoint	0.35 ± 0.22	−0.27 ± 0.14	−0.26 ± 0.16
Coherence (U)	35°C timepoint	0.50 ± 0.08	0.46 ± 0.05	0.36 ± 0.02
	8°C timepoint	0.47 ± 0.04	0.43 ± 0.05	0.37 ± 0.04

The values indicate the means ± SE. ^ indicates significant difference from control. * indicates significant difference from warm water immersion.

### CIVD bins vs. non-CIVD bins

Comparing bins with CIVD using the LDF criteria to general cutaneous vasoconstriction bins with no CIVD yielded increases in nVLF (P = 0.051, *η* ^2^ = 0.399) and nHF (P = 0.021, *η* ^2^ = 0.249) power and trended toward decreases in nLF (P = 0.094, *η* ^2^ = 0.188) power in bins with CIVD (Table 4). TFA gain during CIVD did not yield differences across frequency bins compared to cutaneous vasoconstriction alone (Table 5).

**TABLE 4:** Comparisons of mean spectral powers of LDF signals in cold water immersion (8°C) timepoints in which no CIVD was observed (absent) vs. when a CIVD was observed (occurred). Panel A depicts values in very low (VLF), low (LF), and high (HF) frequency ranges and Panel B depicts normalized values in the same ranges (nVLF, nLF, and nHF) (n = 6 participants who had distinct CIVD vs. non-CIVD bins during cold water immersion; comparisons are between cold water immersion timepoints for the same participant).

Panel A. Skin – absolute Values				
Probe	CIVD	VLF (flux units^2^)	LF (flux units^2^)	HF (flux units^2^)
Laser-Doppler flux (LDF)	absent	167.94 ± 110.45	217.48 ± 146.76	104.13 ± 68.18
	occurred	215.74 ± 101.99	130.38 ± 53.16	55.29 ± 25.86

Panel B. Skin ‐ normalized values				
Probe	CIVD	nVLF (flux units^2^)	nLF (flux units^2^)	nHF (flux units^2^)
Laser-Doppler flux (LDF)	absent	34.25 ± 4.86	46.56 ± 5.05	19.19 ± 2.51
	occurred	50.09 ± 3.76*	36.42 ± 4.31*	13.50 ± 1.86*

The values indicate the means ± SE. * indicates significant difference from no CIVD (absent).

**TABLE 5:** Transfer function analysis of arterial pressure to skin blood flux variability during partial-hand cold water immersion (8°C) in timepoints in which no CIVD was observed (absent) vs. when a CIVD was observed (occurred). Values are depicted in very low (VLF), low (LF), and high (HF) frequency ranges (n = 6 participants who had distinct CIVD vs. non-CIVD bins during cold water immersion; comparisons are between cold water immersion timepoints for the same participant).

Transfer function analysis	CIVD	VLF	LF	HF
Transfer function gain (flux units/mmHg)	absent	0.23 ± 0.07	0.25 ± 0.07	0.28 ± 0.07
	occurred	0.29 ± 0.06	0.19 ± 0.03	0.13 ± 0.03

The values indicate the means ± SE.

## Discussion

The skin is a complex organ and environmental interface with regional structure and functional adaptations. For example, compared to peripheral hairy skin, glabrous acral skin of the hands and feet contains an additional histological layer in the epidermis (stratum lucidum), different skin appendages (no hair follicles), different sensory afferents (Meissner corpuscles), and an additional blood flow system (AVAs). Thus, it is not surprising that the neural control and regulation of skin blood flow varies by skin area. AVAs are relatively large diameter vessels that are rich in vascular smooth muscle and mediated by noradrenergic mechanisms without a classic active vasodilator system. Standard means of inducing maximal skin blood flow, such as local heating to 42°C in hairy limb skin, are less effective in glabrous acral skin (Metzler-Wilson et al., 2012). An attractive CIVD hypothesis is based on data indicating the presence of α_2c_-adrenergic receptors that are trafficked to the vascular smooth muscle membrane during cold stress to augment norepinephrine-induced vasoconstriction (Chotani and Flavahan, 2011; Filipeanu, 2015); this is also an identified mechanism of vasospasm in Raynaud phenomenon (Wigley and Flavahan, 2016). It is unknown if α_2c_-adrenergic receptors are part of the mechanism of CIVD. One could speculate from mouse data that these receptors augment vascular transduction of the sympathetic signal and then reduce augmentation during α_2c_-adrenergic receptor recycling or by release of a compound from the TRPA1-containing sensory nerve (Aubdool et al., 2014). It is possible that receptor recycling or partial overriding of vasoconstriction could be acting as a “functional sympatholysis” (Remensnyder et al., 1962) process in the skin. To interrogate this possibility, we used a FFT-TFA approach to gain insight into the regulation of vasoconstriction during focal cold water-induced CIVD.

Cutaneous vasoconstriction occurred during both cold water-immersed and control fingers as indexed by initial decreases in CVC and skin temperature (Figures 2–4). This is expected and indicative of a systemic autonomic response which would cause cold-induced vasoconstriction and increases in ABP (Table 1). The vasoconstriction in the immersed fingers was pronounced, indicating that additional local temperature-related factors caused or modulated this sympathetic vasoconstriction. Interestingly, by the end of the cold stress, immersed finger CVC returned to baseline while the skin temperature remained depressed. This rise in CVC and LDF values from initial cooling to final cooling could correlate with the vasodilation - or release of vasoconstriction - of the CIVD response. Approximately 83% of participants (10 of 12) demonstrated a CIVD response with skin temperature criteria in the finger pad, and 67% of participants (8 of 12) were identified using LDF criteria in the nailbed. Non-immersed fingers experienced vasoconstriction but not CIVD. This measurement discrepancy reinforces the need for standardization of the variables used to define the response, as a lack of consistency in these variables has been reported to affect its replicability (O'Brien, 2005).

A unique aspect of the current study is the reporting of concentration of skin erythrocytes moving within the laser Doppler field. The concentration of moving erythrocytes is a component of the LDF signal (Nilsson et al., 1980). In theory, this could provide some insight into the amount of blood in surface blood vessels. For example, during heat stress there is a large increase in skin vascular flow and volume (Deschamps and Magder, 1990). In heat stress, the increase in volume may slow vascular transit times and promote heat exchange. During cold stress, there is likely decreased vascular volume because of a combination of cutaneous vasoconstriction and venoconstriction. Isolated saphenous vein work indicates that cooling augments cutaneous venoconstriction to both α-adrenergic agonists and electrical stimulation (Harker et al., 1994; Harker et al., 1990). We observed decreases in erythrocyte concentration in cold water-immersed but not control fingers. Interestingly, erythrocyte concentration remained low even at the end of cooling when CVC had returned to baseline levels. Another approach linking skin temperature and vascular activities in CIVD is the application of photoacoustic ultrasound (Zhang et al., 2024). Since erythrocyte concentration and combined optical and ultrasonic imaging are more rarely reported, interpretations are more difficult, but these observations could provide unique insights as to a possible relationship between the skin temperature and CVC changes.

Using the FFT and autoregressive approaches, the LF range is thought to contain most of the sympathetic signal, as evidenced by direct neural stimulation, blockade, and end-organ observation (Pagani et al., 1992; Stauss et al., 1998). We observed a reduction in both absolute spectral power and normalized spectral power in the LF range in the finger during cold water immersion compared to warm water immersion. Reductions in spectral power seem counterintuitive during cooling, as sympathetic vasoconstriction would seem to be heightened. It is possible that even if sympathetic outflow was only minimally changed during local cooling, additional α_2c_-adrenergic receptors would be recruited, which would augment the concept of sympathetic vascular transduction (Young et al., 2021). Previous wavelet analyses identified “neurologic bin” differences (Hodges et al., 2019; Hodges et al., 2018; Sera et al., 2020). However, direct comparisons cannot be made because wavelet and FFT bins are not the same and are interpreted differently. We observed decreased nLF spectral power in CIVD bins compared to bins that did not result in CIVD. Combined, we feel confident that the frequency bins that can be correlated to the changes in the sympathetic nervous system are being altered with CIVD. The VLF has been associated with local tissue and vessel factors, but the precise mechanisms are still unclear. When the sympathetic nervous system is pharmacologically inhibited (e.g., human ganglionic blockade via systemic trimethaphan), there is a large increase in glabrous skin blood flow and fall in both VLF and LF spectral power (Wilson et al., 2005). We observed no change in absolute VLF spectral power across timepoints, but there were specific interactions in nVLF spectral power. Both warm water and cold water immersion decreased nVLF spectral power from the air baseline. We did not observe differences between cold immersion fingers, indicating similar or uniform VLF responses in immersed fingers. CIVD increased the nVLF spectral power compared to cold water immersion bins that did not result in CIVD. These data seem to indicate that the increase in nVLF may be part of the mechanism of CIVD or possibly a combination of decrease in nLF and increase in nVLF.

TFA gain was lower during cooling compared to air baseline and warm water immersion in the VLF and to an even greater extent in the LF. We interpret this as ABP not causing as much flow change because of cutaneous vasoconstriction. There were no changes in ABP spectral power, despite an increase in ABP. Thus, it is unlikely that changes in ABP are dramatically affecting the data. TFA gain was not helpful in differentiating between bins with CIVD and those without. In contrast, when the sympathetic nervous system is blocked, ABP pressure fluctuations result in much greater changes in TFA gain in glabrous acral skin (Wilson et al., 2005). Combined, we interpret these findings as indicating that CIVD release of vasoconstriction is likely modest, which is not great enough to alter the pressure-flow relations in the cold water-immersed fingers.

As noted in the Methods, we employed several experimental controls and additional parameter measurements than those standardly reported in the field. First, we simultaneously tested two experimental fingers and a control finger. Also, we used thermoneutral water temperature to allow for a more uniform pre-vasoconstriction starting point. Importantly, we accounted for ABP, which increases during systemic and focal cooling (Cui et al., 2002; Wilson et al., 2007) and is correlated to AVA opening/closing (Walloe, 2016). Additionally, because the skin possesses little intrinsic autoregulatory ability, ABP drives pressure for cutaneous perfusion, unless perfusion pressure is substantially reduced (Durand et al., 2004). Finally, we used TFA to relate LDF to ABP, which allowed for the calculation of gain, phase, and coherence between the two signals in the skin (Wilson et al., 2005).

Our analysis approach was both a strength and a limitation to our experiments. Wavelets are very efficient signal processing transformations because they have both time and frequency components. To gain this efficiency, wavelets give up some frequency resolution, especially in the lower frequency ranges. We used FFT, with its high frequency resolution across time series bins, to compare bins with and without CIVD. Thus, we were less efficient in signal processing but likely have better lower-frequency precision compared to previous wavelet analyses in this area. During vasoconstriction, spectral power often drops; even if driven by the sympathetic nervous system, flow amplitude can be decreased to such an extent that it makes interpretations more difficult. We do not currently have a fix for this issue, but more low flow data collection and analysis will improve understanding of the pressure-flow relations in these conditions. Another limitation was that the control finger did not experience hydrostatic forces; the primary rationale for this was the need to measure ABP in the contralateral finger. We felt the tradeoff was worth the measurements gained. All subjects participated in the protocols, but we were able to match CIVD and non-CIVD segments within the same recording in only 6 subjects. We handled these data sets as separate analysis and reported both sets of results. We did not power our studies for sex comparisons, but we do not think this significantly alters our interpretations, as recent evidence suggests that sex does not play a critical role in modulating CIVD (Weller et al., 2024).

CIVD has been postulated to protect against local cold injury and to improve dexterity and tactile sensitivity (Alba et al., 2019; Cheung, 2015; Daanen, 2003). It is possible that understanding the mechanisms of CIVD release of cold-induced vasoconstriction may provide insights into treatment targets for cold-induced vasospasms occurring in Raynaud phenomenon (Kadian-Dodov, 2023; Stringer and Femia, 2018). The mechanisms underpinning CIVD have not been fully resolved other than being related to the sympathetic control and regulation of AVAs in glabrous skin (Walloe, 2016). It is possible that a substance released from TRPA1-containing sensory afferents regulates and attenuates this sympathetic-induced cutaneous vasoconstriction (Aubdool et al., 2014). Our data could be interpreted as supporting this type of “functional sympatholysis”. Although it is difficult to parse out these potential mechanisms, previous researchers (Hodges et al., 2018) have interpreted their data as supporting sympathetic withdrawal. However, it is difficult to know when there is sympathetic withdrawal vs. decreased sympathetic vascular transduction. This question will likely not be directly addressed until someone directly measures the skin sympathetic nerve activity of the median nerve during a CIVD. Nevertheless, oscillations in skin blood flow appear to provide unique insights into potential mechanisms of vasoconstriction and periodic release of vasoconstriction that characterize this cutaneous reflex.

## Conclusion

Despite being identified nearly 100 years ago, the underlying mechanisms for CIVD are still unclear, and the optimal means of quantification have proved somewhat elusive. We utilized a sympathetic neurovascular approach to address aspects of these open research questions. We did not support our hypothesis that CIVD would have increased transfer function gain compared to vasoconstriction without dilation. This indicates that dual increases in arterial blood pressure and skin blood flux occur in a similar magnitude. We supported our hypothesis that FFT and TFA would provide an altered CIVD signature compared to cold segments without a CIVD and that CIVD would have a significant effect on VLF and LF components of FFT. Our data identified alterations in both sympathetic and local signal components, where local components are increasing and sympathetic components are decreasing during CIVD. This may indicate that local factors are operating against the sympathetic tone in a “functional sympatholysis” manner which modestly affects sympathetic vascular transduction on a periodic basis during cold water immersion.

## Data Availability

The raw data supporting the conclusions of this article will be made available by the authors, without undue reservation.
